# In Vivo Demonstration of the Superior Replication and Infectivity of Genotype 2.1 with Respect to Genotype 3.4 of Classical Swine Fever Virus by Dual Infections

**DOI:** 10.3390/pathogens9040261

**Published:** 2020-04-03

**Authors:** Yu-Liang Huang, Kuo-Jung Tsai, Ming-Chung Deng, Hsin-Meng Liu, Chin-Cheng Huang, Fun-In Wang, Chia-Yi Chang

**Affiliations:** 1 Animal Health Research Institute, Council of Agriculture, Executive Yuan, 376 Chung-Cheng Road, Tansui, New Taipei City 25158, Taiwan; ylhuang@mail.nvri.gov.tw (Y.-L.H.); krtsai@mail.nvri.gov.tw (K.-J.T.); mcdeng@mail.nvri.gov.tw (M.-C.D.); hmliu@mail.nvri.gov.tw (H.-M.L.); 2 Council of Agriculture, Executive Yuan, No. 37 Nanhai Road, Taipei 10014, Taiwan; cch@mail.coa.gov.tw; 3 School of Veterinary Medicine, National Taiwan University, No. 1, Section 4, Roosevelt Road, Taipei 10617, Taiwan

**Keywords:** classical swine fever virus, genotype, virus shift, viral replication, dual infections

## Abstract

In Taiwan, the prevalent CSFV population has shifted from the historical genotype 3.4 (94.4 strain) to the newly invading genotype 2.1 (TD/96 strain) since 1996. This study analyzed the competition between these two virus genotypes in dual infection pigs with equal and different virus populations and with maternally derived neutralizing antibodies induced by a third genotype of modified live vaccine (MLV), to simulate that occurring in natural situations in the field. Experimentally, under various dual infection conditions, with or without the presence of maternal antibodies, with various specimens from blood, oral and fecal swabs, and internal organs at various time points, the TD/96 had consistently 1.51−3.08 log higher loads than those of 94.4. A second passage of competition in the same animals further widened the lead of TD/96 as indicated by viral loads. The maternally derived antibodies provided partial protection to both wild type CSFVs and was correlated with lower clinical scores, febrile reaction, and animal mortality. In the presence of maternal antibodies, pigs could be infected by both wild type CSFVs, with TD/96 dominating. These findings partially explain the CSFV shift observed, furthering our understanding of CSFV pathogenesis in the field, and are helpful for the control of CSF.

## 1. Introduction

Classical swine fever virus (CSFV) is the etiological agent of classical swine fever (CSF), which is a highly contagious disease of swine. The CSFV is an enveloped positive-stranded RNA virus belonging to the genus *Pestivirus* of the family *Flaviviridae* [1]. The genome of CSFV is approximately 12.3 kb in length and contains a single open reading frame encoding for a polyprotein of 3,898 amino acids, which is flanked by 5′ and 3′ non-translated regions (NTR). The translated polyprotein is processed by cellular and viral proteases to the mature viral proteins of four structural (C, E^rns^, E1, and E2) and eight nonstructural proteins (N^pro^, p7, NS2, NS3, NS4A, NS4B, NS5A, and NS5B) [2].

The CSFV strains are divided into three genotypes, each with three to four subtypes—1.1, 1.2, 1.3, and 1.4; 2.1, 2.2, and 2.3; and 3.1, 3.2, 3.3, and 3.4—by analyzing three genomic regions: 5′-UTR, E2, and NS5B [3,4]. Genotype 1 comprises most of the historical strains including vaccine strains. Genotype 2 contains the most currently globally prevalent strains over the last two decades. Genotype 3 contains most of the strains with restricted distribution [3,5,6,7]. In recent years, there has been a shift in CSFV genotypes in the field, from genotypes 1 and 3 to genotype 2, which was observed in Europe and Asia [8,9,10,11]. The mechanisms for this shift remain unclear. In Taiwan, two CSFV populations coexist, namely the historical strain of genotype 3.4 (94.4 strain) prior to the 1920s and the newly invading strain of genotype 2.1 (TD/96 strain) since 1994, the latter of which became dominant in 1996 [12,13]. This means that in the field, pigs could be infected by two different genotypes of CSFV. In pig infections, the dominance of one strain, such as TD/96, over another strain, such as 94.4, could cause potential problems in the diagnosis, pathogenesis and epidemiological studies and control of CSF in the field, if not given special attention. From a clinical point of view, this replication advantage of genotype 2.1 could likely mask the detection, isolation and more aspects of genotype 3.4. The situation is further complicated by the routine use of modified live vaccines (MLV) to prevent and control CSF, in which all MLV strains used nowadays in different countries belong to genotype 1, a third genotype [3,14]. The MLVs offer protection from field viruses of different genotypes [14,15,16]. However, several factors, including viral loads of vaccines, routes and ages of vaccination, and co-presence of other pathogens, can interfere with the vaccine’s efficacy [14,16,17].

To deepen our understanding of the characteristics of diverse CSFV genotypes, it is important to elucidate the mechanism of the virus shift in the field. Previous study revealed that the newly invading genotype 2.1 replicated more efficiently than genotype 3.4 did both in vitro and in vivo [18]. To further the understanding of pathogenesis that occurs in dual infections of CSFV, this study analyzed the competitions of the viruses of the two genotypes in co-infected pigs with equal and different virus populations without neutralizing antibodies and also in co-infected pigs with maternally derived neutralizing antibodies induced by MLV of a third genotype, with the goal of experimentally simulating the natural situations in the field.

## 2. Results

### 2.1. Clinical Manifestation

Pigs in Group 1 (co-infected_P1) were inoculated with equal amounts of the TD/96/TWN strain (designated TD/96 and belonging to genotype 2.1) and the 94.4/IL/94/TWN strain (designated 94.4 and belonging to genotype 3.4) simultaneously. Pigs in Group 2 (co-infected_P2) were inoculated with whole blood taken from a pig of Group 1 at 12 dpi. Pigs in Group 3 (co-infected with Ab) were inoculated with equal amounts of the TD/96 strain and the 94.4 strain simultaneously, born from a sow vaccinated with LPC vaccine, in which the maternal antibody response was in decline.

**Figure 1:**
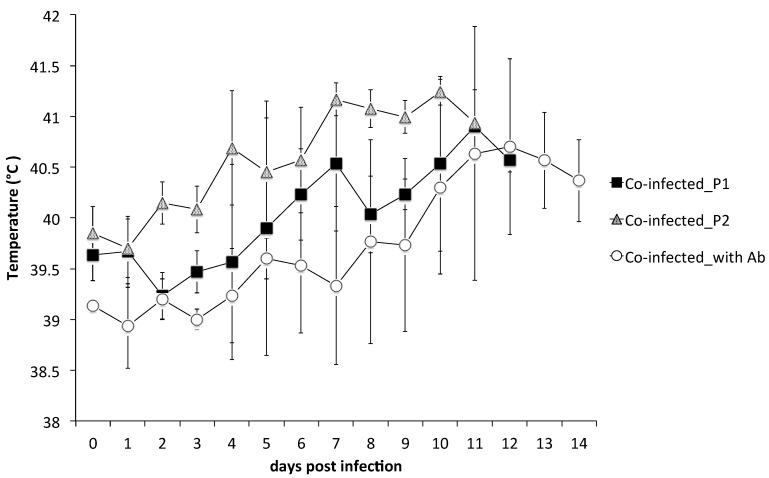
Body temperatures of pigs co-inoculated with classical swine fever viruses of two genotypes. Pigs in the group co-infected_P1 (square) were inoculated with TD/96 strain (genotype 2.1) and 94.4 strain (genotype 3.4). Pigs in the group co-infected_P2 (triangle) were inoculated with whole blood taken from a pig of group co-infected_P1 at 12 days post-infection. Pigs in the group co-infected with Ab (circle) were born from a sow vaccinated with LPC vaccine and were inoculated with the two virus strains. Each point represents the mean and standard deviation of the three pigs in the same group.

Clinical scores and temperature records of the experimental pigs are shown in Figure 1 and Table 1. The clinical signs were most numerous and significantly severe in co-infected_P2 (second passage of competition) pigs (Group 2; average maximum clinical score: 19.33 ± 0.58) and the first febrile reaction was detected as early as 2 or 3 days post-infection (dpi). The highest clinical score of Group 2 was the result of the pigs being inoculated with higher viral loads of the TD/96 strain than the pigs of the other two groups were (Table 1).

Those pigs in co-infected_P1 (first passage of competition) were less severe (Group 1; average maximum clinical score: 15.67 ± 1.53), and first febrile reaction was detected at 4 to 6 dpi. Clinical signs of those pigs co-infected with the presence of maternal antibodies developed more slowly and were significantly less severe (Group 3; average maximum clinical score: 4.67 ± 0.58) and all pigs survived until the end of experiment. First febrile reaction in pigs in Group 3 was detected at 6 to 12 dpi.

The febrile profile shown in Figure 1 was largely compatible with those of the averaged clinical scores shown in Table 1. In other words, Group 2, having higher clinical scores, also had higher febrile reactions, while Group 3, having lower clinical scores, also had lower febrile reaction.

### 2.2. Virus Titration of Viremia for Co-Infected_P2 Pigs Inoculation

Co-infected_P2 pigs were inoculated with whole blood taken from a pig of the co-infected_P1 group at 12 dpi (Group 2, Table 1). The virus titers of the TD/96 strain and the 94.4 strain from viremia at 12 dpi of a co-infected_P1 pig were 10^8.3^ TCID_50_/mL (tissue culture infectious dose 50%) and 10^5.87^ TCID_50_/mL, respectively, determined by CSFV genotype-specific monoclonal antibodies (mAbs) (Table 2).

### 2.3. Cross-Neutralizing Antibodies against Three Genotypes of CSFVs

The role of maternal antibodies during dual infections was further investigated (Table 3). The pigs’ sera of co-infected_P1 (Group 1) and co-infected_P2 (Group 2) at 0 dpi to the end of the experimental period showed no neutralizing antibodies against genotypes 1.1 (LPC/AHRI strain), 2.1 (TD/96 strain) or 3.4 (94.4 strain), consistent with the more severe clinical scores and animal mortalities at 9–13 dpi observed in both groups (Table 1).

The neutralizing antibodies against the LPC strain in the sera of co-infected pigs with maternal antibodies (Group 3) before inoculation indicated that these pigs were in a declining phase of maternal antibody response (data not shown). Sera of co-infected pigs at 0–6 dpi did show cross-neutralizing antibodies against three genotypes (Table 3), consistent with the much milder clinical scores seen in this group. A critical time window was noted at 6–8 dpi, when the cross-neutralizing antibody titers against TD/96 and 94.4 CSFVs dropped from log_2_ 2.3–2.8 to < 2.0 (Table 3), and yet the animals survived until 14 dpi (Table 1). The neutralizing antibody titers against the LPC strain were significantly higher than those against the TD/96 strain and the 94.4 strain at 0 and 2 dpi and significantly higher than those against the 94.4 strain at 4 and 6 dpi. There was no significant difference between the neutralizing antibody titer against the TD/96 strain and the 94.4 strain. From 8 or 10 dpi to 14 dpi, sera of co-infected with Ab pigs did not show detectable neutralizing antibodies against any of the three genotypes (Table 3).

### 2.4. Viral Loads in Bloods

**Figure 2:**
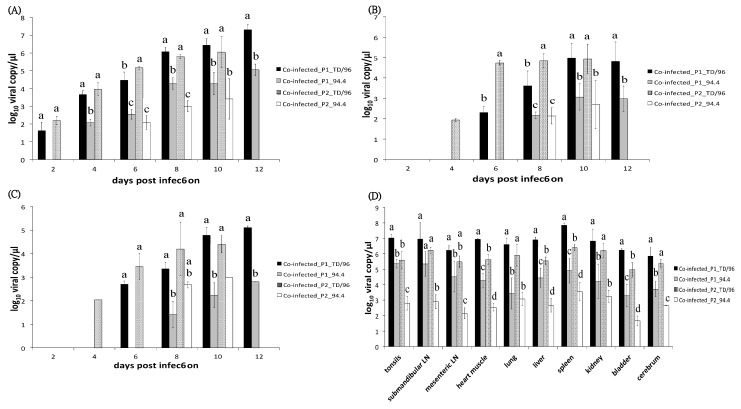
Comparison of viral loads of co-infected_P1 (Group 1) and co-infected_P2 pigs (Group 2). Viral loads in (**A**) blood, (**B**) oral swabs, (**C**) fecal swabs, and (**D**) organs of co-infected_P1 and co-infected_P2 pigs were quantified by reverse transcription multiplex real-time polymerase chain reaction. The data represent the mean and standard deviation from three pigs. Values with different superscript letters, a–d, among the four groups of samples at the same dpi (**A–C**) or the same organ (**D**) indicate a statistically significant difference (*p* < 0.05) from each other. The superscript letter “a” indicates the highest viral load and “d” indicates the lowest viral load among the compared groups, while “ab” indicates a viral load in between categories “a” and “b”. No significant differences exist between values containing the same letter. The absence of a superscript letter indicates no statistical analysis due to only one sample of a group or only one group within the same dpi.

**Figure 3:**
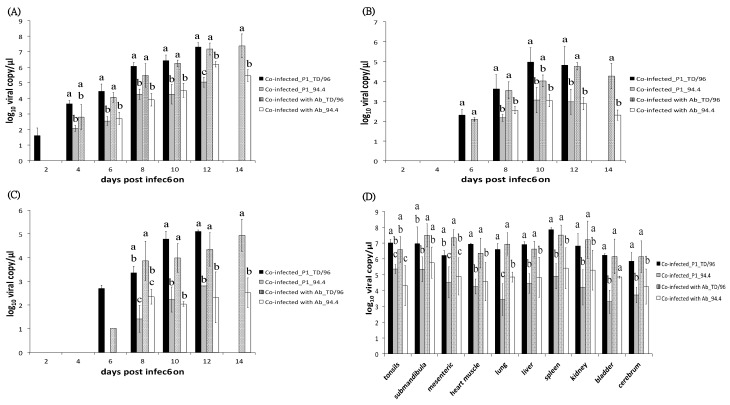
Comparison of viral loads of co-infected_P1 (Group 1) and co-infected with Ab pigs (Group 3). Viral loads in (**A**) blood, (**B**) oral swabs, (**C**) fecal swabs, and (**D**) organs of co-infected_P1 and co-infected with Ab pigs were quantified by reverse transcription multiplex real-time polymerase chain reaction. The data represent the mean and standard deviation from three pigs. Values with different superscript letters, a–d, among the four groups of samples at the same dpi (**A–C**) or the same organ (**D**) indicate a statistically significant difference (*p* < 0.05) from each other. The superscript letter “a” indicates the highest viral load and “d” indicates the lowest viral load among the compared groups, while “ab” indicates a viral load in between categories “a” and “b”. No significant differences exist between values containing the same letter. The absence of a superscript letter labeled indicates no statistical analysis due to only one sample of a group or only one group within the same dpi.

Primary viremia of TD/96 strain was first detected in both co-infected_P1 pigs and co-infected_P2 pigs at 2 dpi, and in co-infected with Ab pigs at 4 dpi, earlier than that of 94.4 strain, which was first detected in co-infected_P1 pigs at 4 dpi and in both co-infected_P2 pigs and co-infected with Ab pigs at 6 dpi (Figure 2A and Figure 3A, Table 1). The viral loads of the TD/96 strain were always significantly higher than those of the 94.4 strain (*p* < 0.05) (Figure 2A and Figure 3A) in all the co-infected pigs. The viral loads of the TD/96 strain were on average 1.89, 2.93, and 1.51 log higher than that of the 94.4 strain at all dpi in co-infected_P1 pigs, co-infected_P2 pigs and co-infected with Ab pigs, respectively. Comparing the three groups, the co-infected_P2 pigs had the highest lead of TD/96 at all dpi; the viral loads were above 3 log higher than that of the 94.4 strain at 4 and 6 dpi. The highest lead of TD/96 was observed at 4 dpi, when it was on average 3.96 log higher than that of the 94.4 strain. No lead of TD/96 was above 3 log in co-infected_P1 pigs or co-infected with Ab pigs.

### 2.5. Viral Loads in Secretions and Excretions

In co-infected_P2 pigs, the TD/96 strain was first detected in oral swabs and fecal swabs at 4 to 6 dpi, whereas in co-infected_P1 pigs and co-infected with Ab pigs, the TD/96 strain first presented at 6 to 8 dpi (Table 1). The 94.4 strain was first detected in oral swabs and fecal swabs at 8 to 10 dpi in all co-infected pigs, except one co-infected_P2 pig that died at 9 dpi, in which the virus was not detected (Table 1).

In oral swabs, the viral loads of the TD/96 strain were always significantly higher than those of the 94.4 strain in all the co-infected pigs (*p* < 0.05) (Figure 2B and Figure 3B), with only one exception at 10 dpi of co-infected with Ab pigs. The viral loads of the TD/96 strain were on average 1.88, 3.08 and 1.59 log higher than that of the 94.4 strain at all dpi in co-infected_P1 pigs, co-infected_P2 pigs and co-infected with Ab pigs, respectively. Comparing the three groups, the co-infected_P2 pigs had the highest lead of TD/96 at all dpi, in which the viral loads were above 3 log higher than that of the 94.4 strain at 6 and 8 dpi. The highest lead of TD/96 was observed at 6 dpi, when it was on average 4.74 log higher than that of the 94.4 strain. No lead of TD/96 was above 3 log in co-infected_P1 pigs or co-infected with Ab pigs.

In fecal swabs, the viral loads of the TD/96 strain were always significantly higher than those of the 94.4 strain in all co-infected pigs (*p* < 0.05) (Figure 2C and Figure 3C), with only one exception at 8 dpi of co-infected_P2. The viral loads of the TD/96 strain were on average 2.49, 2.7 and 1.84 log higher than that of the 94.4 strain at all dpi in co-infected_P1 pigs, co-infected_P2 pigs and co-infected with Ab pigs, respectively. Comparing the three groups, the co-infected_P2 pigs had the highest lead of TD/96 at all dpi; the highest lead of TD/96, with on average 3.45 log higher than that of the 94.4 strain, was observed at 6 dpi. No lead of TD/96 was above 3 log in co-infected_P1 pigs or co-infected with Ab pigs.

### 2.6. Viral Loads in Visceral Organs

The viral loads of the TD/96 strain were consistently significantly higher than those of the 94.4 strain in most tested organs (*p* < 0.05) (Figure 2D and Figure 3D) in all pigs. The viral loads of the TD/96 strain were on average 2.39, 3.00, and 2.09 log higher than those of the 94.4 strain in co-infected_P1 pigs, co-infected_P2 pigs, and co-infected with Ab pigs, respectively. Comparing the three groups, the co-infected_P2 pigs had the highest lead of TD/96 in most tested organs; the viral loads were above 2.5 log higher than that of the 94.4 strain. The highest lead of TD/96 was observed in lymph nodes, where it was on average 3.33 log higher than that of the 94.4 strain.

## 3. Discussion

The shift in CSFV populations in the field, from genotypes 1 and 3 to genotype 2, was observed worldwide [8,9,10,11,12]. However, the mechanisms responsible for the shift remain unclear. A previous study hypothesized that genotype 2 had higher genetic diversity than genotypes 1 and 3 did, which might explain why it is the most prevalent endemic situation [19]. Three hypotheses were proposed: First, virus strains of genotype 2.1 may have higher replication efficiency than the genotype 3.4 strains in pigs; second, the strains of genotype 2.1 may have higher affinity and competitiveness to cellular receptors than those of genotype 3.4; and third, the strains of genotype 2.1 may have better ability than those of genotype 3.4 to escape from antibody neutralization induced by the attenuated lapinized vaccine strain LPC of genotype 1.1, which has been used to protect pigs against the 3.4 strains since the 1950s in Taiwan [12]. To test the first two hypotheses, Huang et al. [18] allowed two viruses belonging to genotypes 2.1 and 3.4, respectively, to compete in vivo and in vitro, and the results revealed that the virus of genotype 2.1 replicated more efficiently than that of genotype 3.4. To further explore and to simulate the field situation, this study analyzed the competitions of the viruses of the two genotypes in co-infected pigs for two passages without neutralizing antibodies (Groups 1 and 2). Moreover, we also examined the dynamics of virus replication and disease development of the infected pigs in the presence of maternally derived neutralizing antibodies induced by LPC vaccine (Group 3). To the best of our knowledge, this study is the first attempt to test the competitions of CSFV in this way.

The new CSFV strain of genotype 2.1 has higher replication efficiency than the historical genotype 3.4 strain in pigs. Indeed, when given equal opportunity to compete in the same animal for the first passage of competition, the genotype 2.1 CSFV (represented by TD/96) had 2.43 log (8.3–5.87) higher TCID_50_ titer over that of genotype 3.4 (represented by 94.4) in the blood (Group 2 inoculum, Table 1). This advantage of TD/96 in blood was also amply supported by quantifications of viral loads using quantitative reverse transcription multiplex real-time polymerase chain reaction (RT-MRT-PCR) (Table 2). Under various dual infection conditions with or without the presence of maternal antibodies, the TD/96 strain had consistently 1.51−3.08 log higher loads than those of 94.4. The 2.43 log TCID_50_ replication advantage of TD/96 in the first passage of competition in an animal body falls within the range of 1.51–3.08 log, as estimated by RT-MRT-PCR. Given a second passage of competition, the lead of TD/96 was widened further. The TD/96 strain was first detected in oral swabs and fecal swabs of co-infected_P2 pigs 2–4 days earlier than in those of co-infected_P1 pigs. On the other hand, the 94.4 strain was first detected in viremia of co-infected_P2 pigs 2 days later than in those of co-infected_P1 pigs, despite the two groups having been inoculated with similar amounts of the 94.4 strain (10^6^ TCID_50_/mL in co-infected_P1 pigs and 10^5.87^ TCID_50_/mL in co-infected_P2 pigs). The results revealed that when the viruses of genotype 2.1 are dominant in the field, the viruses replicate more efficiently and shed earlier than the viruses of genotype 3.4 do. Therefore, the genotype of CSFV in the pig population shifted from genotype 3.4 to 2.1. Given further passages of dual infection in pigs, the competition edge of genotype 2.1 would likely lead to the disappearance of genotype 3.4 in pigs.

Colostrum maternal antibodies offer partial protection for dually infected pigs, despite the genotype difference of the LPC vaccine virus (of genotype 1.1) from those of genotype 2.1 and 3.4 of dually infected viruses (Group 3). The TD/96 and 94.4 strains were first detected in the blood of co-infected with Ab pigs 2 days later than in co-infected_P1 pigs, although pigs in these two groups were inoculated with the same amounts of the TD/96 and 94.4 strains. The maternal antibodies protection was ineffective when the genotypic heterologous neutralizing titer dropped to below 1:4 during dpi 8−14 (Table 3), for febrile reactions were observed after 10 dpi (Figure 1). This finding suggests that the protection offered by the maternal antibodies is not limited to the initial engagement to neutralize incoming virus but also allows for the host to launch immune responses before the system is overwhelmed.

Antigenic variations among various genotypes of CSFVs certainly render the currently available vaccines more effective in neutralizing historical viruses, while allowing the newly invading virus to escape. This hypothesis has been amply addressed in several previous studies [20,21,22,23]. The neutralizing antibodies of co-infected with Ab pigs against the LPC/AHRI strain were higher than those against the TD/96 strain and the 94.4 strain. However, there was no significant difference between the neutralizing antibodies against the TD/96 strain and those against the 94.4 strain. This evidence supported previous studies that antibodies induced by live virus neutralize genotypically homologous strains better than heterologous strains [17,24,25]. In co-infected with Ab pigs, similarly to Group 1, the TD/96 strain was shed by infected pigs earlier than the 94.4 strain was. In addition, the viral loads of the TD/96 strain were significantly higher than those of the 94.4 strain. The results indicated that the presence of maternally derived antibodies induced by modified live virus of genotype 1.1 might not influence the competition between viruses of genotypes 2.1 and 3.4. Previous study demonstrated that the LPC vaccine could offer pigs protection from challenges of field viruses of genotypes 2.1 and 3.4 [26]. This study revealed that when pigs or piglets infected by CSFV field viruses of genotypes 2.1 and 3.4 were vaccinated, the vaccine’s efficacy was interfered with, and also that when the maternally derived antibodies declined in piglets before vaccination, the pigs produced lower neutralizing antibodies and had delayed clinical signs. However, the CSFVs were detected in those infected pigs, in which the genotype 2.1 viruses were released earlier and replicated more efficiently than did genotype 3.4 viruses. These results may suggest that the newly invading strains dominated in the field under vaccination.

Contact infection may present another aspect of virus competition. To investigate how the competition occurs between viruses of genotypes 2.1 and 3.4, in this study we employed the intramuscular route for the co-infection. This route can ensure that both viruses enter the hosts with the same amount of virus simultaneously to allow comparison of their replication efficiency and pathogenicity. However, when the virus is introduced through natural routes, the influencing factors could be more numerous and much complicated. To further explore the natural situations and to evaluate the possible effect of the superinfection exclusion phenomenon of CSFV [27] in the field in terms of the competence of these two genotypes, cohabitation infection with 2.1 virus infected pigs and 3.4 virus infected pigs is warranted.

In conclusion, examining the competition of the historical and newly invading genotypes of CSFV in co-infected pigs with different virus populations and with maternally derived neutralizing antibodies revealed that the new CSFV genotype 2.1 replicates more efficiently, at 1.51–3.08 log higher than that of the historical genotype 3.4. The maternally derived antibodies provide partial protection to both wild type CSFVs and correlate with lower clinical scores, febrile reaction, and animal mortality. In the presence of maternal antibodies, pigs could be infected by both wild type CSFVs, with the genotype 2.1 dominating. These results could further our understanding of the prevalence of genotype 2 in the field, which is widely observed in Asia and Europe. This is the first time that the higher replication capacity of genotype 2.1 than that of genotype 3.4 has been demonstrated in vivo with this design.

## 4. Materials and Methods

### 4.1. Cells and Viruses

Porcine kidney-15 (PK-15) cells were maintained in minimum essential medium supplemented with 10% fetal bovine serum and incubated at 37 °C in 5% CO_2_. The three CSFV strains used in this study, comprised the two representative CSFV strains TD/96 and 94.4 and an attenuated lapinized vaccine strain LPC/AHRI, were propagated in the PK-15 cells [3,12,13].

### 4.2. mAbs Specific for CSFV

Three mAbs against CSFVs were used in this study. The mAbs T6 and L71 were produced by the Animal Health Research Institute, Taiwan, and the mAb WH303 by the Animal and Plant Health Agency, the United Kingdom. The mAb T6 recognizes the TD/96 strain of genotype 2.1 but not the 94.4 strain of genotype 3.4. In contrast, the mAb L71 recognizes the 94.4 strain but not the TD/96 strain [18]. The mAb WH303 reacts with most CSFV strains tested [28], including the three strains used in this study.

### 4.3. Experimental Infections

Six 4-week-old specific pathogen-free (SPF) pigs were randomly separated into two groups of three pigs: Groups 1 and 2 (Table 1). Pigs in Group 1 (co-infected_P1) were inoculated intramuscularly with 1 mL of the TD/96 strain and 1 mL of the 94.4 strain simultaneously, each at a virus amount of 10^6^ TCID_50_/mL, to ensure that both strains could enter the hosts simultaneously. The concept of the Group 1 co-infection experiment design was similar to that described in Group 3 of Huang et al. [18] and was repeated here, in separate pigs, for comparison with Groups 2 and 3 (see below). Pigs in Group 2 (co-infected_P2) were inoculated intramuscularly with 1 mL of whole blood taken from a pig of Group 1 at 12 dpi. This inoculum contained TD/96 10^8.3^ TCID_50_ and 94.4 10^5.87^ TCID_50_ as titrated later (Table 1). Group 3 (co-infected with Ab) included three 4-week-old pigs born from a sow vaccinated with LPC vaccine, in which the maternal antibody response was in decline. These animals were chosen in order to examine the effect of antibody drop on co-infection. These pigs were inoculated intramuscularly with 1 mL of the TD/96 strain and 1 mL of the 94.4 strain simultaneously, as in Group 1, at 10^6^ TCID_50_/mL. The three groups were housed separately in three negative air-pressure isolation units. For animal welfare reasons, pigs were euthanized when they were moribund and unable to stand up. All surviving pigs were euthanized at 14 dpi, the end of the experimental period. This animal experiment was approved by the Institutional Animal Care and Use Committee of the Animal Health Research Institute (Approval number A02040).

### 4.4. Clinical Signs, Body Temperature, and Sampling Procedures

Rectal temperature was recorded daily during the experimental period. Fever was defined as a temperature higher than 40 °C. For evaluation of clinical signs, the ten parameters described by Mittelholzer et al. [29] were scored from 0 to 3 to represent normal to severe CSF symptoms. The scores of each pig were summed into a total score for each day. Blood, oral swabs, and fecal swabs were collected prior to inoculation at 0 dpi and then at 2-day intervals post infection. Swabs were weighed before and after sampling to normalize the viral loads. Each swab was immersed in 2 mL of phosphate buffered saline (PBS) and centrifuged at 3,000 × *g* for 10 min, and the harvested supernatant was stored at −70 °C. Necropsies were performed after euthanasia or death, and tissue samples of tonsil, submandibular and mesenteric lymph nodes, heart muscle, lung, liver, spleen, kidney, bladder, and cerebrum were collected from all pigs.

### 4.5. Virus Titration

Ten-fold serial diluted blood (inoculum of Group 2) at 12 dpi from a pig of co-infected_P1 was added into eight wells each of 96-well plates duplicated and seeded with PK-15 cells. Whether the cells were infected was observed using indirect fluorescent assay (IFA) at 72 hours post infection (hpi). The mAbs T6 and L71 were used for virus titration. One 96-well plate was stained with mAb T6, which recognizes the TD/96 strain but not the 94.4 strain; the other 96-well plate was stained with mAb L71, which recognizes the 94.4 strain but not the TD/96 strain [18]. Virus titers were calculated as TCID_50_ using the Reed-Muench method [30].

### 4.6. Quantitative Reverse Transcription Multiplex Real-Time Polymerase Chain Reaction (RT-MRT-PCR)

Viral RNAs were extracted using the QIAamp^®^ Viral RNA Mini Kit (QIAGEN, Hilden, Germany). The specific RT-MRT-PCR was performed to detect and genotype CSFV as described by Huang et al. [31], who demonstrated no inter-genotypic cross-reactivity among different CSFV strains using the universal primers and specific TaqMan probes for each of the three genotypes, genotypes 1, 2, and 3. The viral loads, determined by the RT-MRT-PCR, are expressed as log viral genome copies/μL.

### 4.7. Cross-Neutralizing Antibodies against Three Genotypes of CSFV

The genotype-specific neutralizing antibodies were cross-neutralized with sera from pigs at 0 dpi (before inoculation) to 14 dpi (or the end of the experimental period) against CSFV strains of genotypes 1.1 (LPC/AHRI strain), 2.1 (TD/96 strain) and 3.4 (94.4 strain). Two-fold serial diluted 56 °C heat-inactivated sera were mixed with equal volumes of 100 TCID_50_ of the viruses, incubated at 37 °C for 1 h, and subsequently transferred to PK-15 cells in 96-well plates. The starting dilution of each serum was 1:4. At 72 hpi, the cells were fixed and stained for the presence CSFV antigen by the IFA. Neutralizing titer is the log_2_ of the antibody dilution factor (reciprocal of dilution) when 50% of the wells are protected from infection.

### 4.8. Indirect Fluorescent Assay (IFA)

The inoculated cells in 96-well plates were fixed with 10% formaldehyde at room temperature for 10 min and washed three times with PBS. Each mAb against CSFV was diluted 1:100 in PBS, and 50 μL of the diluted mAb was added per well (mAb T6 for virus titration of the TD/96 strain and mAb L71 for virus titration of the 94.4 strain; mAb WH303 for detection of cross-neutralizing antibodies). The cells were then incubated at 37 °C for 1 h and washed three times with PBS. Fluorescein isothiocyanate-conjugated goat anti-mouse IgG (Jackson ImmunoResearch Laboratories, West Grove, PA, USA) diluted 1:100 in PBS was added, 50 μL per well. The cells were incubated at 37 °C for 1 h and then washed three times with PBS. Fluorescence of the stained cells was observed under a fluorescence microscope (Olympus Imaging America, Center Valley, PA, USA).

### 4.9. Statistical Analysis

Differences in the values between two groups and among various groups were statistically analyzed using the Student’s t-test and one-way analysis of variance (ANOVA), respectively. ANOVA was combined with the Duncan multiple range test. The statistical analysis was carried out in Statistical Analysis System (SAS) Enterprise Guide 7.1 (SAS Institute Inc., Cary, NC, USA). Mean differences were considered statistically significant when the *p*-value was <0.05.

## Figures and Tables

**Table 1:** Clinical scores and virus detection of individual pigs co-inoculated with classical swine fever viruses.

Inoculated Group	Time (dpi) of the First Observation of Fever	Maximum Clinical Score	Time (dpi) of Death	Time (dpi) of the First Detection of Virus in Blood		Time (dpi) of the First Detection of Virus in Oral Swabs		Time (dpi) of the First Detection of Virus in Fecal Swabs	
				TD/96^†^	94.4	TD/96	94.4	TD/96	94.4
Group 1: Co-infected_P1	4	17	13	2	4	6	8	6	8
(TD/96 10^6^ TCID_50_ + 94.4 10^6^ TCID_50_)	6	16	13	2	4	6	8	8	10
	6	14 Average 15.67 ± 1.53^b*^	13	2	4	6	8	6	8
Group 2: Co-infected_P2	2	19	9	2	6	4	ND^§^	4	ND
(blood of group 1 pig containing TD/96 10^8.3^ TCID_50_ and 94.4 10^5.87^ TCID_50_)	3	20	12	2	6	4	8	6	8
	2	19 Average 19.33 ± 0.58^a^	11	2	6	6	8	6	8
Group 3: Co-infected with Ab	12	5	14^¶^	4	6	6	8	8	8
(TD/96 10^6^ TCID_50_ + 94.4 10^6^ TCID_50_)	10	5	14^¶^	4	6	6	8	6	8
	6	4 Average 4.67 ± 0.58^c^	14^¶^	4	6	8	8	8	8

^†^ TD/96 and 94.4 indicate the TD/96/TWN and 94.4/IL/94/TWN strains, respectively. * Values with different superscript letters, a-c, among the three groups of samples at the same dpi indicate a statistically significant difference (*p* < 0.05) from each other. The superscript letter “a” indicates the highest viral load and “c” indicates the lowest viral load among the compared groups. ^¶^ The pig was euthanized at the end of the experiment. ^§^ ND: not detected.

**Table 2:** Viral loads in blood from a pig of the co-infected_P1 group at 12 dpi.

Viral Loads	Log		Methods	
	TD/96^†^	94.4	TD/96	94.4
Viral titer (TCID_50_/mL)	8.3	5.87	By IFA^*^ using mAb T6 specific for TD/96	By IFA using mAb L71 specific for 94.4
Viral genome (copies/μL)	7.64	5.23	By RT-MRT-PCR^¶^ using specific TaqMan probe for TD/96	By RT-MRT-PCR using specific TaqMan probe for 94.4

^†^ TD/96 and 94.4 indicate the TD/96/TWN and 94.4/IL/94/TWN strains, respectively. * IFA indicates indirect fluorescent assay. ^¶^ RT-MRT-PCR indicates reverse transcription multiplex real-time polymerase chain reaction.

**Table 3:** Cross-neutralizing antibodies of three pigs co-inoculated with classical swine fever viruses with maternally derived neutralizing antibodies (Group 3).

Time (dpi) of the Collected Pig Sera	Cross-Neutralizing Antibodies Against (log_2_)			Time (dpi) of the Collected Pig Sera	Cross-neutralizing Antibodies Against (log_2_)		
	LPC^†^	TD/96	94.4		LPC	TD/96	94.4
0	4.5	2.5	2.5	8	2.5	<2	<2
	4.5	3.5	3.5		3.5	<2	<2
	5	3.5	2.5		<2	<2	<2
	Average 4.7 ± 0.3^a*^	Average 3.2 ± 0.6^b^	Average 2.8 ± 0.6^b^				
2	4	2	2	10	<2	<2	<2
	4.5	3	3		<2	<2	<2
	5	3.5	2.5		<2	<2	<2
	Average 4.5 ± 0.5^a^	Average 2.8 ± 0.8^b^	Average 2.5 ± 0.5^b^				
4	3	2.5	2	12	<2	<2	<2
	5	3.5	3		<2	<2	<2
	5	3	2.5		<2	<2	<2
	Average 4.3 ± 1.2^a^	Average 3 ± 0.5^ab^	Average 2.5 ± 0.5^b^				
6	3	2.5	2	14	<2	<2	<2
	4.5	3.5	3		<2	<2	<2
	5	2.5	2		<2	<2	<2
	Average 4.2 ± 1^a^	Average 2.8 ± 0.6^ab^	Average 2.3 ± 0.6^b^				

^†^ LPC, TD/96 and 94.4 indicate the LPC/AHRI, TD/96/TWN and 94.4/IL/94/TWN strains, respectively. * Values with different superscript letters, a–b, among the three groups of samples at the same dpi indicate a statistically significant difference (*p* < 0.05) from each other. The superscript letter “a” indicates the highest viral load, and “b” indicates the lowest viral load among the compared groups, while “ab” indicates a viral load in between categories “a” and “b”. No significant difference exists between values containing the same letter. The absence of a superscript letter indicates no statistical analysis.
